# A newly developed deep learning-based system for automatic detection and classification of small bowel lesions during double-balloon enteroscopy examination

**DOI:** 10.1186/s12876-023-03067-w

**Published:** 2024-01-02

**Authors:** Yijie Zhu, Xiaoguang Lyu, Xiao Tao, Lianlian Wu, Anning Yin, Fei Liao, Shan Hu, Yang Wang, Mengjiao Zhang, Li Huang, Junxiao Wang, Chenxia Zhang, Dexin Gong, Xiaoda Jiang, Liang Zhao, Honggang Yu

**Affiliations:** 1 Department of Gastroenterology, Renmin Hospital of Wuhan University, Wuhan, China; 2 Key Laboratory of Hubei Province for Digestive System Disease, Renmin Hospital of Wuhan University, Wuhan, China; 3 Hubei Provincial Clinical Research Center for Digestive Disease Minimally Invasive Incision, Renmin Hospital of Wuhan University, Wuhan, China; 4 School of Computer Science, Wuhan University, Wuhan, China

**Keywords:** Double-balloon enteroscopy, Artificial intelligence, Small bowel

## Abstract

**Background:**

Double-balloon enteroscopy (DBE) is a standard method for diagnosing and treating small bowel disease. However, DBE may yield false-negative results due to oversight or inexperience. We aim to develop a computer-aided diagnostic (CAD) system for the automatic detection and classification of small bowel abnormalities in DBE.

**Design and methods:**

A total of 5201 images were collected from Renmin Hospital of Wuhan University to construct a detection model for localizing lesions during DBE, and 3021 images were collected to construct a classification model for classifying lesions into four classes, protruding lesion, diverticulum, erosion & ulcer and angioectasia. The performance of the two models was evaluated using 1318 normal images and 915 abnormal images and 65 videos from independent patients and then compared with that of 8 endoscopists. The standard answer was the expert consensus.

**Results:**

For the image test set, the detection model achieved a sensitivity of 92% (843/915) and an area under the curve (AUC) of 0.947, and the classification model achieved an accuracy of 86%. For the video test set, the accuracy of the system was significantly better than that of the endoscopists (85% vs. 77 ± 6%, *p* < 0.01). For the video test set, the proposed system was superior to novices and comparable to experts.

**Conclusions:**

We established a real-time CAD system for detecting and classifying small bowel lesions in DBE with favourable performance. ENDOANGEL-DBE has the potential to help endoscopists, especially novices, in clinical practice and may reduce the miss rate of small bowel lesions.

**Supplementary Information:**

The online version contains supplementary material available at 10.1186/s12876-023-03067-w.

## Background

Double-balloon enteroscopy (DBE) is important for managing small bowel disease [1]. Different from capsule endoscopy (CE), DBE not only serves as a diagnostic tool but allows for tissue sampling and therapeutic intervention [2, 3]. DBE has a real-time image reading mode. However, there are inherent oversight and distraction risks in the use of this mode due to its time-consuming and technically demanding nature. Additionally, diagnosis is influenced by endoscopist experience variability. It has been previously reported that the first DBE procedures yielded false-negative results in 20–23.8% of patients [4–7]. The lesion types missed during these procedures include ulcers, diverticulum, tumours, and vascular lesions [6]. As immediate repeat inspection is not routine for DBE, missed lesions are unlikely to be detected during the same procedure. This will delay the detection and treatment of bleeding lesions, which increases the risk of recurrent bleeding and repeat examinations [6].

Artificial intelligence (AI) has become a strong focus of interest in clinical practice, owing to its potential to reduce diagnostic errors and manual workload [8–12]. Convolutional neural networks (CNNs) have been used for big data analysis of medical images [9, 13–21]. CNN-based algorithms have displayed outstanding performance in gastrointestinal endoscopy that is comparable to or even superior to that of experts [22–26]. AI may assist in diagnosis during endoscopic examinations by automatically detecting, characterizing, measuring, and localizing various lesions. Computer-aided diagnostic (CAD) systems could improve the examination quality and diagnostic accuracy in gastrointestinal endoscopy, helping clinicians formulate therapeutic strategies and prognosis predictions [27–30]. Recently, there has been overwhelming literature supporting the crucial role of CNNs on CE to automatically recognize classify, and localize small bowel abnormities [31–43]. However, there is little research on the detection and classification of DBE.

In this study, we aim to develop a CNN-based system for automatically detecting multiclass small bowel lesions in both DBE images and videos. This system might increase the diagnostic yield for small bowel lesions that are responsible for gastrointestinal bleeding and reduce the burden of repeat DBE.

## Methods

### Study design and datasets

#### Study design

This study aims to build a deep learning system named ENDOANGEL-DBE for detecting and classifying small bowel lesions in DBE. This is a retrospective, single-centre, and diagnostic study.

ENDOANGEL-DBE is composed of two models. Model 1 is responsible for detection, and Model 2 is responsible for classification (diverticulum, protruding lesion, erosion & and ulcer, angioectasia). ENDOANGEL-DBE will give results on the lesion location and type in real time during DBE.

The individual performance of the two models was tested by evaluating two image test sets separately and ENDOANGEL-DBE was tested through the evaluation of 65 videos and compared to 8 human observers, including 4 novices and 4 experts.

#### Datasets

All images and videos for training and testing were retrospectively collected from Renmin Hospital of Wuhan University (RHWU). Images that revealed the characteristics of small bowel lesions were included in this study.

The Model 1 training set included 5201 images, with 4201 abnormal images as positive samples and 1000 normal images as noise, from 463 patients from November 1st, 2016 to October 30th, 2020 (Supplementary Table 1).

The Model 1 image test set included 915 abnormal images from 98 patients and 1318 normal images from 89 patients from November 1st, 2020 to June 15th, 2021 (Supplementary Table 1).

The Model 2 training set included 3021 abnormal images from 389 patients from November 1st, 2016 to October 30th, 2020. A total of 4171 lesion images were abstracted for Model 2 training according to the standard answer, i.e., the consensus of three experts (Supplementary Table 2).

The Model 2 image test set included 915 abnormal images from 98 patients, which were the same as the abnormal images in the Model 1 image test set. A total of 1422 lesion images were abstracted for Model 2 training according to the standard answer (Supplementary Table 2).

The Video test set was collected from June 16th, 2021 to November 12th, 2021. The Video test set contained 65 video clips from 30 consecutive patients, including 2 with diverticulum, 30 with protruding lesions, 25 with erosions and ulcers, and 8 with angioectasia. The average length of the videos was 66.29 ± 21.14 seconds. Each video clip was used to extract consecutive images at a rate of 25 frames per second (Supplementary Table 3).

All images were obtained from DBE (EN-450 T5, EN-530 T; Fujifilm, Tokyo, Japan) and video systems (VP-4450HD; Fujifilm, Tokyo, Japan). There was no overlap of patients between the training and test sets.

### Development of the CAD system

#### Image processing

All the black frames in the original images were automatically cropped according to pixel changes. Moreover, the training set was enhanced by rotating, transforming, resizing, and cropping.

#### Experts reached a consensus on the standard answer of the images

Three experts (L Zhao, A Yin, and F Liao) reached a consensus to obtain the standard answer for training and testing. If more than two experts came to the same conclusion for a given image, the conclusion was the standard answer; otherwise, the experts discussed their findings and reached a consensus. The experts, with more than 200 cases of DBE experience, were all from RHWU. They labeled images by bounding each lesion with the smallest rectangular box that enclosed the lesion through an online annotator [44], and the labels were the standard answer for training and testing Model 1. Then, they classified all images into diverticulum, protruding lesion, erosion & ulcer, and angioectasia as the standard answer for training and testing Model 2.

Protruding lesions included: polyps, nodules, epithelial tumours, submucosal tumours, and venous structures. Erosions and ulcers were classified into the same category. Erosions, ulcers, and diverticulum included lesions of various aetiologies. Angioectasia included Yano–Yamamoto classification types 1a and 1b.

#### Construction of the CAD system

**Fig. 1:**
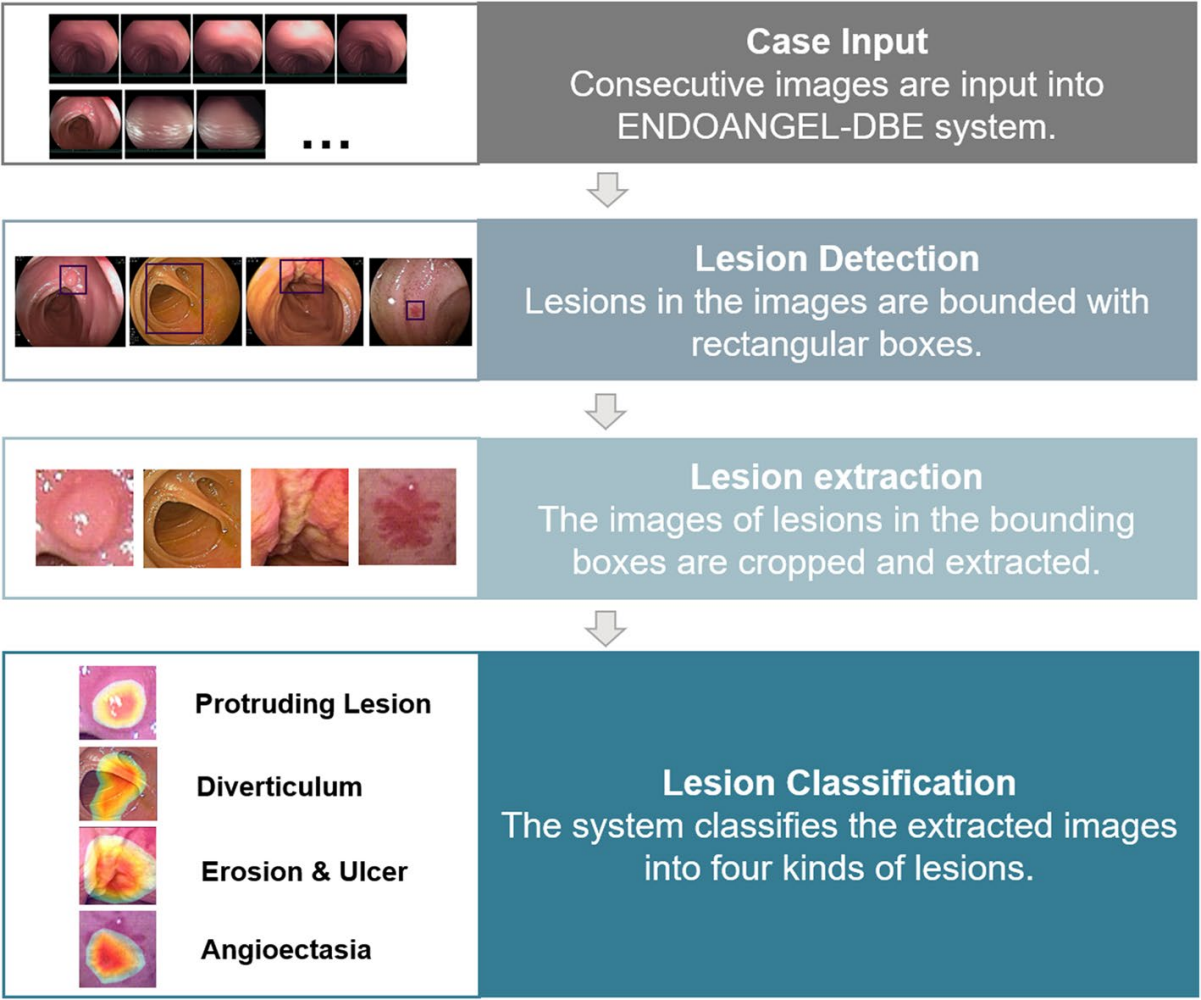
Processing flow diagram of ENDOANGEL-DBE

The CAD system consisted of two CNNs. The structure of ENDOANGEL-DBE is shown in Fig. 1. You only look once (YOLO) [45] was used for detection (Model 1). YOLO is a region-based object detector that uses a single CNN to detect lesion regions. YOLO has been trained for lesion detection in digestive endoscopic examinations and is widely used in studies for its fast and accurate detection [46–48]. ResNet-50 [49], a residual learning framework with good generalizability, was used to build Model 2. The residual blocks of ResNet-50 utilize skip connections to avoid vanishing gradients. The dataset was trained in Google’s TensorFlow. Mode l tuning was used for training. The model tuning parameters are shown in Supplementary Table 4. Dropout, data augmentation, and early stopping with patience of val_loss were used to lower the overfitting risk (Supplementary Tables 5 to 6).

The threshold value of Model 1 was set to 0.02 according to the ROC curve. Because Model 2 was a four-type classification model, the label with the highest threshold value and with a threshold value ≥0.25 among the four classification labels assigned to the target image was the final classification that the model outputs.

#### Training and testing

**Fig. 2:**
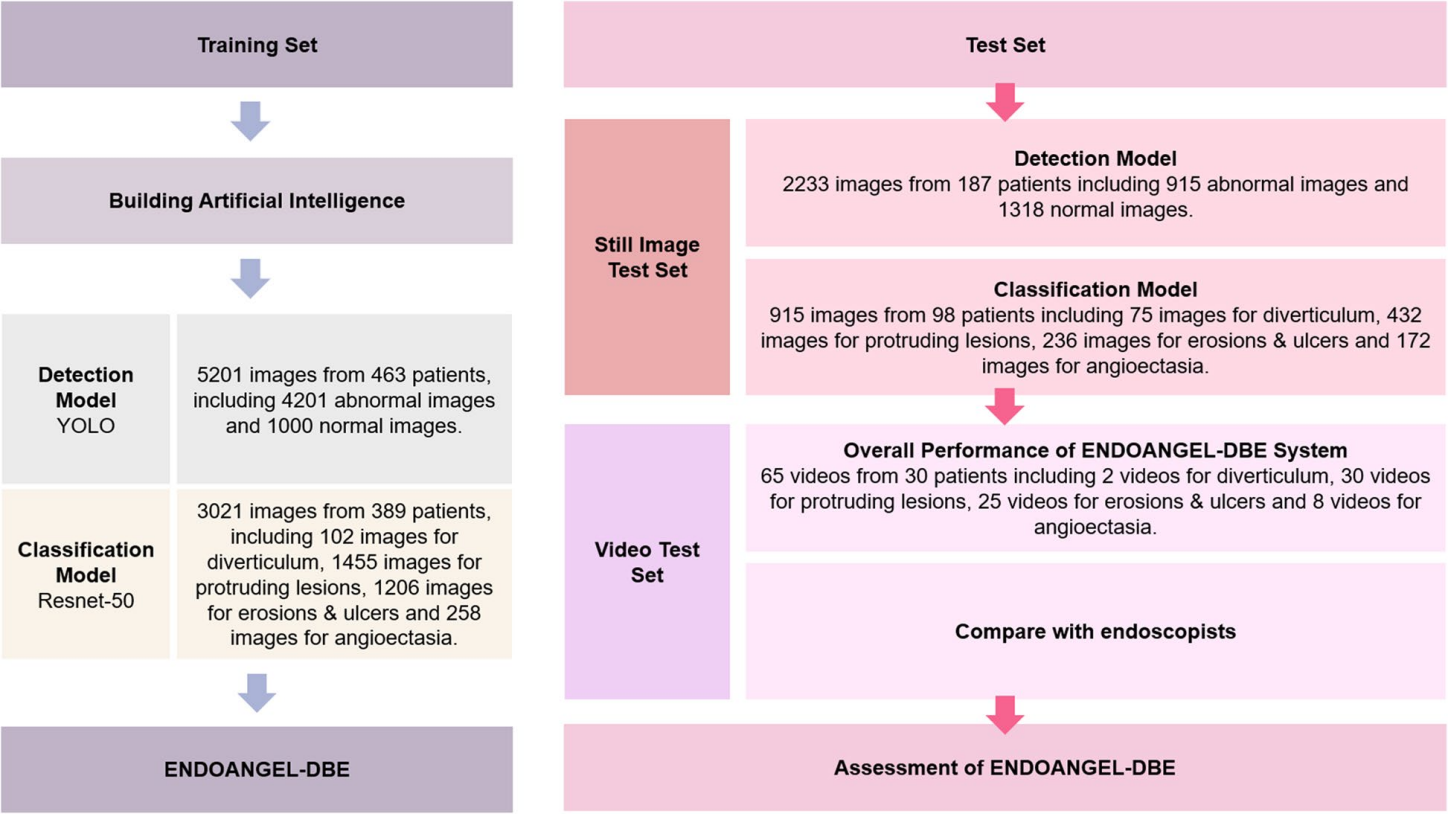
Flow chart

The training and testing flow chart is shown in Fig. 2 and the dataset distribution is shown in Supplementary Tables 1 to 3. We evaluated the individual performance of the two models with the image test set and the overall performance of ENDOANGEL-DBE with the video test set. In addition, ENDOANGEL-DBE performance was compared with endoscopists’ performance for the video test set.

### Assessment with image test sets

#### Assessment of YOLO

The area under the receiver operating characteristic (ROC) curve (AUC), sensitivity, and specificity were used to evaluate the performance of Model 1. The standard answer of the three experts, as mentioned above, was obtained using an online annotator. Model 2 used a bounding box to annotate lesions, and as long as there was one box bounding lesion, the model’s detection of the current image was considered correct.

#### Assessment of ResNet-50

In the image test set, lesion images abstracted from original images according to the standard answer obtained by the three experts mentioned above were used to assess the performance of Model 2. Accuracy, sensitivity, and specificity were used to evaluate the models.

### Assessment with the retrospective video test set and comparison with endoscopists

We evaluated the overall performance of the proposed system on the video test set. Model 1 detected lesions with bounding boxes and Model 2 classified the boxed lesions, and the output classification results were recorded. Three experts provided standard answers for this video test set and we evaluated ENDOANGEL-DBE’s performance in terms of accuracy, sensitivity, and specificity. The videos were cropped by 25 frames per second, and the lesions were boxed by Model 1 and then input into Model 2 for classification. The models provided a diagnosis every second, and the final result of a video was obtained from the largest proportion of all the diagnoses from the video.

Four novices with less than 10 cases of DBE experience and an additional 4 experts with more than 200 cases of DBE experience participated in a human and machine contest. The 4 experts in the video test were different from the 3 experts who developed the standard answers. They diagnosed the same 65 videos independently. The test in ENDOANGEL-DBE was performed 8 times to compare the results with those 8 endoscopists.

### Statistical analysis

Continuous variables are shown as the mean and standard deviation. McNemar’s test was used for comparisons between ENDOANGEL-DBE and the endoscopists. All calculations were performed using SPSS 26 (IBM, Chicago, Illinois, USA).

### Ethics

The Ethics Committee approved this study at RHWU. The Ethics Committees waived informed consent in this retrospective study. Patient information was hidden during training and testing.

## Results

### Performance of ENDOANGEL-DBE for images

The AUC of Model 1 for the image test set was 0.947. The ROC curve is shown in Supplementary Fig. 1. The sensitivity of Model 1 in detecting lesions was 92% and the specificity was 93%. Ninety-eight out of 1318 normal images in the image test set were recognized as abnormal images, and 72 out of 915 abnormal images had no box bounding lesion. Normal mucosa, mucus, feces, light spots, dim spots, bubbles, and dark view induced false-positive results. Normal mucosa was the primary contributor to false-positive images. Most of the false-negative images were misjudged owing to dark view, lesions resembling the surrounding mucosa, and lesions with small flat haematin bases (Table 1).

**Table 1:** False positive images & false negative images analysis in the image test set

	Misjudged reasons	Number of images
**False positive images**	Normal mucosa	53
	Mucus & faeces	18
	Light spot & dim & bubbles	24
	Dark view	3
**False negative images**^**a**^	Diverticulum	33
	Erosion & Ulcer	15
	Protruding lesion	19
	Angioectasia	5

^a^ Most of the false negative images were misjudged by the following factors: dark view, lesions that are similar to surrounding mucosa and few of lesions with small flat hematin base

Typical images for classification are shown in Supplementary Fig. 2. The overall accuracy of Model 2 was 86%. The sensitivity of classifying protruding lesions was 93%, which was the highest among the four common lesions, and the lowest sensitivity was 80% (erosions & ulcers and diverticulum). ENDOANGEL-DBE also achieved high performance in classifying angioectasia in the image test set (85%). The specificity of classifying diverticulum was 99%.

### Performance of ENDOANGEL-DBE for videos

There were 65 videos in the video test set. The detection sensitivity was 100% in the per-case analysis and 93% in the per-frame analysis. The total classification accuracy of ENDOANGEL-DBE was 85%. The number of correct cases that ENDOANGEL-DBE and human observe for different classes of lesions is shown in Table 2. The performance of ENDOANGEL-DBE for the video test set is shown as a confusion matrix in Supplementary Fig. 3.

**Table 2:** The number of correct cases diagnosed by endoscopists and ENDOANGEL-DBE in video test set. The results of endoscopists are shown as averages ± standard deviations

	ENDOANGEL-DBE	Endoscopists	Experts	Novices
**Diverticulum**	2	2.0 ± 0.0	2.0 ± 0.0	2.0 ± 0.0
**Protruding lesion**	27	27.6 ± 0.9	27.8 ± 0.4	27.5 ± 1.1
**Erosion& ulcer**	20	15.6 ± 4.6	13.5 ± 1.9	17.8 ± 5.5
**Angioectasia**	6	4.9 ± 1.5	4.5 ± 1.1	5.3 ± 1.7

### Human and machine comparison

The total accuracy of endoscopists was 77%. The overall performance of ENDOANGEL-DBE was superior to that of endoscopists (*p* < 0.01). Twenty out of 25 erosion & ulcer cases were diagnosed correctly by ENDOANGEL-DBE, and it had more correct cases than endoscopists in this class. The number of cases that ENDOANGEL and endoscopists diagnosed correctly in diverticulum and protruding lesions were comparable. In addition, the overall performance of ENDOANGEL-DBE was superior to that of novices (73%, *p* < 0.01). ENDOANGEL diagnosed more correct cases than the novices did in classifying erosion &ulcer and angioectasia. The overall performance of ENDOANGEL was comparable to the experts in classification (81%; *p* = 0.253). And it diagnosed more correct cases than experts did in classifying angioectasia. The number of correct cases that ENDOANGEL and experts diagnosed correctly was comparable in classifying the other three lesion classes (Table 2). A demonstration video is shown in Video 1.

**Fig. 3:**
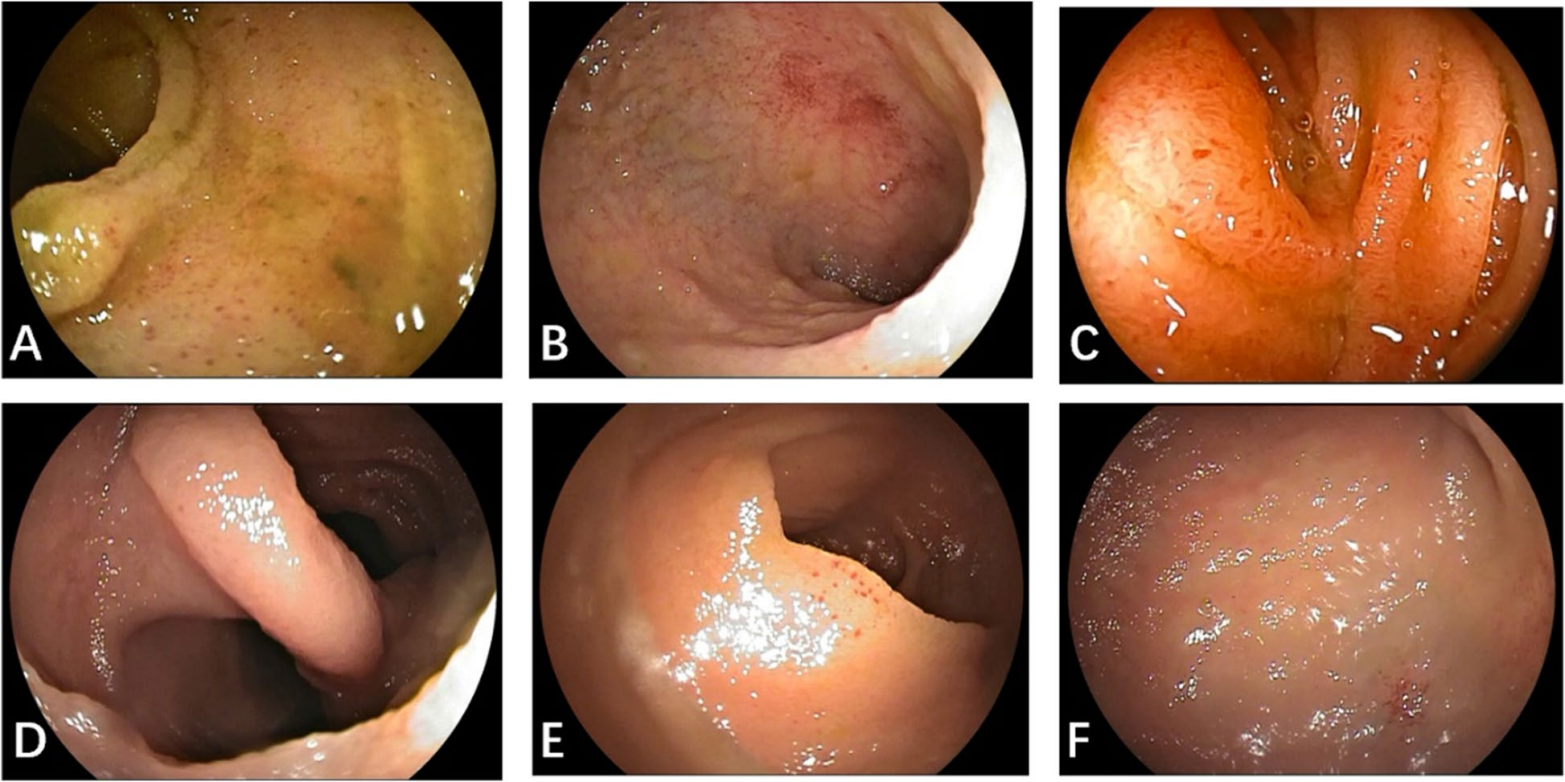
Misdiagnosed cases of machine or endoscopists. **A** Erosions misdiagnosed as angioectasia by novices because bile affects observation. **B** small erosions misdiagnosed as angioectasia by novices. **C** Angioectasia with red background misdiagnosed as erosion&ulcer by machine and novices. **D** Pedunculated polyps in the lumen during peristalsis misdiagnosed as diverticulum by machine. **E** small angioectasia misdiagnosed as erosion& ulcer by machine. **F** angioectasia in the edge of view misdiagnosed as erosion& ulcer by machine

In this video test, some erosion and angioectasia cases were confused. ENDOANGEL-DBE misdiagnosed three angioectasia cases as erosions and one erosion as angioectasia. One protruding lesion was misdiagnosed as a diverticulum because of peristalsis (Fig. 3).

## Discussion

The promising performance of CNN-based AI algorithms on CE images inspired us to explore their utility in image analysis in the field of DBE images.

In this study, we developed a CNN-based system to automatically recognize and classify small bowel lesions. This is the first study to develop a CAD system and evaluate it with both DBE images and videos. The CAD system performed similar to experts’ evaluations and better than novices. It might be a useful tool, to improve diagnostic yield in DBE, especially for novices. ENDOANGEL-DBE has the potential to reduce missed lesions, interoperator variability, and improve diagnostic accuracy for common small bowel lesions. Another potential application of ENDOANGEL-DBE is in DBE training. Automatic detection and classification CNN programs can assist in image reading training via real-time feedback, which may shorten the training time and accelerate experience accumulation for trainees. Furthermore, the classification model of this CAD system will facilitate further exploitation towards an automatic diagnosis and reporting system for DBE examination.

Automatic detection assists in reducing the miss rate of four common lesions instead of a single type, and classification will be finished after detecting them, which is one of the advantages of our study compared with previous studies [50–52]. Our system has reached a high accuracy in detecting and classifying small bowel lesions. To reduce false-positive cases, we used normal images as noise in the training set of the detection model, and we achieved satisfactory results. Notably, we found that ENDOANGEL-DBE’s diagnostic precision of erosion & ulcer was the lowest among the four classes of lesions for both the image and video test sets. Most of the misdiagnosed cases were small, red, and spot lesions, especially those with a red background. The targeted collection of small erosion and angioectasia images for further training improvement is necessary to improve ENDOANGEL-DBE diagnostic performance. In the video test set, dark view and red background were the main reasons for false-positive results for ENDOANGEL-DBE. Transfer learning will be used to decrease false-positive cases in further studies. In this video test, the main reason for endoscopists’ misdiagnosis is that the lesions are small, the background in the video is red in tone and bile can also affect observation. We found that ENDOANGEL-DBE diagnosed more correct cases than experts in diagnosing angioectasia in the video test set. Since there were only eight cases of angioectasia included in this video test set, we need further validation in larger test sets to make the results comparable.

Recent studies have shown that the sensitivity of AI in single disease classification under device-assisted enteroscopy has reached 88.5–97% [50–52]. Their training set included more images than our study, but the number of recruited cases in these studies was less than that in ours. Increasing the number of recruited cases will improve the generalizability of the system. These studies were only based on images and our study also assessed ENDOANGEL-DBE’s performance with videos. Miguel Martins et al. [51] developed a model recognizing erosions and ulcers from normal images, whose sensitivity was higher than ours. Since they did not use an independent test set, the images in their test set and training set might come from the same cases, and using such a test set might lead to higher results than those in the real world. These studies aim to detect one single type of lesions, but our system can detect and classify multiple types of lesions. AI systems for automatic detection and classification under CE achieved accuracy of 88.2–100% [31, 33, 34, 53]. These studies contained very large training sets and reached a high detection accuracy. Referring to the above research, we could attempt to use multiple binary classification models or enrich our training sets to further improve our system.

Several limitations of this study must be acknowledged. First, this is a single-centre retrospective study, and only endoscopists from RHWU participated. We should conduct a larger test among endoscopists from different hospitals to compare the performance of the CAD system and humans. We will also plan a multicentre prospective clinical trial to assess its performance in daily clinical routines. Second, the standard answer of this study is expert consensus instead of pathology. The model development was strongly dependent on the consensus of the three experts, which were humans. This study mainly focuses on endoscopic diagnosis. A comprehensive diagnostic system combining pathological and clinical results will be constructed in future studies. Additionally, to further improve our system, model selection, dataset revision, and cross-validation will be used for model training. Third, to make the comparison results fair, the endoscopists were told that the test cases were one of the four types of lesions. However, this might lead to a higher diagnostic accuracy of endoscopists than that in clinical diagnosis. A further comparison between endoscopists with and without AI in unclipped videos is needed to investigate the influence of AI. Fourth, lesions might be missed when multiple different lesions appear at the same time during examinations because of the distance from endoscopy and lesion size. ENDOANGEL-DBE needs further training using multiple lesion images for clinical use. This system will be improved to make diagnoses and assess their relevance to bleeding in future studies. Furthermore, we can develop a system with a positioning function in the future, which can pinpoint the location of lesions. The system can be trained for the assessment of lesion size and the detection of obscure small intestinal bleeding. It could also be used to write an automatic report, which will lessen the administrative workload of endoscopists.

## Conclusions

In conclusion, we developed a novel computer-aided diagnostic system for small bowel lesions in DBE. This CAD system can assist especially novice endoscopists in increasing their diagnostic yield.

### Supplementary Information

**Additional file 1.**

**Additional file 2:** **Video 1.** This is a demonstration video of how the system ENDOANGEL-DBE works. Real-time bounding boxes will appear when the system detects a lesion, and its class is shown on the left side of the screen. ENDOANGEL-DBE will generate a heatmap when the image is stable.

## Data Availability

Individual de-identified participant data that underlie the results reported in this article and study protocol will be shared for investigators after article publication. To gain access, data requesters will need to contact the corresponding author.

## Abbreviations

DBE: Double-balloon enteroscopy
CAD: Computer-aided diagnostic
AUC: Area under the curve
CE: Capsule endoscopy
AI: Artificial intelligence
CNN: Convolutional neural network
RHWU: Renmin Hospital of Wuhan University
VAI: VGG Image Annotator
ROC: Receiver operating characteristic
DAE: Device-assisted enteroscopy
