# Supplementary material for: A newly developed deep learning-based system for automatic detection and classification of small bowel lesions during double-balloon enteroscopy examination
Source: BMC Gastroenterol. 2024 Jan 2;24:10. doi: 10.1186/s12876-023-03067-w (PMC10759410; doi:10.1186/s12876-023-03067-w)
Supplement: Supplementary file 1 — Additional file 1. [file 12876_2023_3067_MOESM1_ESM.docx]

### Supplementary Methods and Materials

**Numerical calculation method**

Sensitivity is the true positive predictions/ the total true positive images; specificity is the true negative predictions/ the total true negative images; accuracy is the accurate prediction of images/ the total images.

**Supplementary Table 1. Distribution of training and testing set for Model 1.**

|  | **Normal** | **Abnormal** | | | |
| --- | --- | --- | --- | --- | --- |
| **Number of images** |  | diverticulum | protruding lesion | erosion& ulcer | angioectasia |
| **Training set** | 1000 | 137 | 2087 | 1643 | 334 |
| **Testing set** | 1318 | 75 | 432 | 236 | 172 |

**Supplementary Table 2. Distribution of training and testing set for Model 2.**

|  | **diverticulum** | **protruding lesion** | **erosion& ulcer** | **angioectasia** |
| --- | --- | --- | --- | --- |
| **Training set** |  |  |  |  |
| Number of images | 102 | 1455 | 1206 | 258 |
| Number of annotations^*^ | 102 | 1778 | 1699 | 592 |
| **Testing set** |  |  |  |  |
| Number of images | 75 | 432 | 236 | 172 |
| Number of annotations^*^ | 76 | 522 | 333 | 491 |

*Lesion images abstracted from the original images according to the gold standard.

**Supplementary Table 3. Distribution of video test set.**

|  | **diverticulum** | **protruding lesion** | **erosion& ulcer** | **angioectasia** |
| --- | --- | --- | --- | --- |
| **Number of videos** | 2 | 30 | 25 | 8 |

**Supplementary Table 4. Model tuning parameters**

| **Model** | **Training** | **Inference** |
| --- | --- | --- |
| YOLO | learning rate | score, nms iou |
| ResNet-50 | learning rate, decay, momentum | - |

**Supplementary Table 5. Data augmentation methods and parameters**

| model | width shift range | height shift range | vertical flip | horizontal flip | augment  hsv | mosaic |
| --- | --- | --- | --- | --- | --- | --- |
| YOLO | 0.2 | 0.2 | True | True | 0.5 | True |

**Supplementary Table 6. Data augmentation methods and parameters**

| model | width shift range | height shift range | rotation range | fill mode | vertical flip | horizontal flip |
| --- | --- | --- | --- | --- | --- | --- |
| ResNet-50 | 0.2 | 0.2 | 90 | constant | True | True |


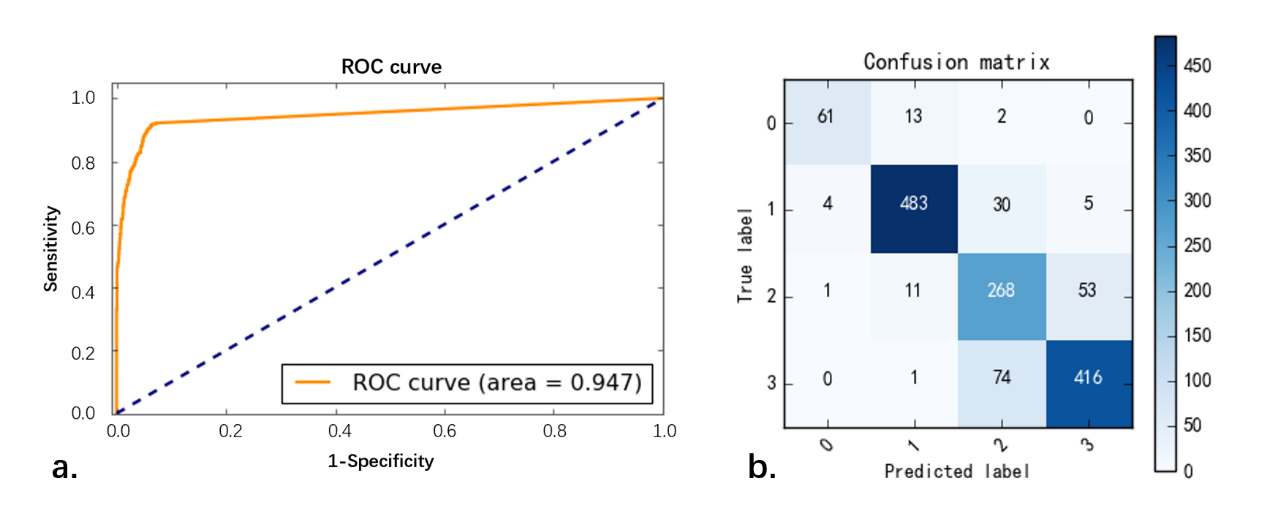


**Supplementary Figure 1. Results of two models on image test set.** a. ROC Curve of Model1 in the image test set. b. Confusion Matrix of Model2 in the image test set: “0” represents “diverticulum”, “1” represents “protruding lesions”, “2” represents “erosion& ulcer” and “3” represents “angioectasia”.


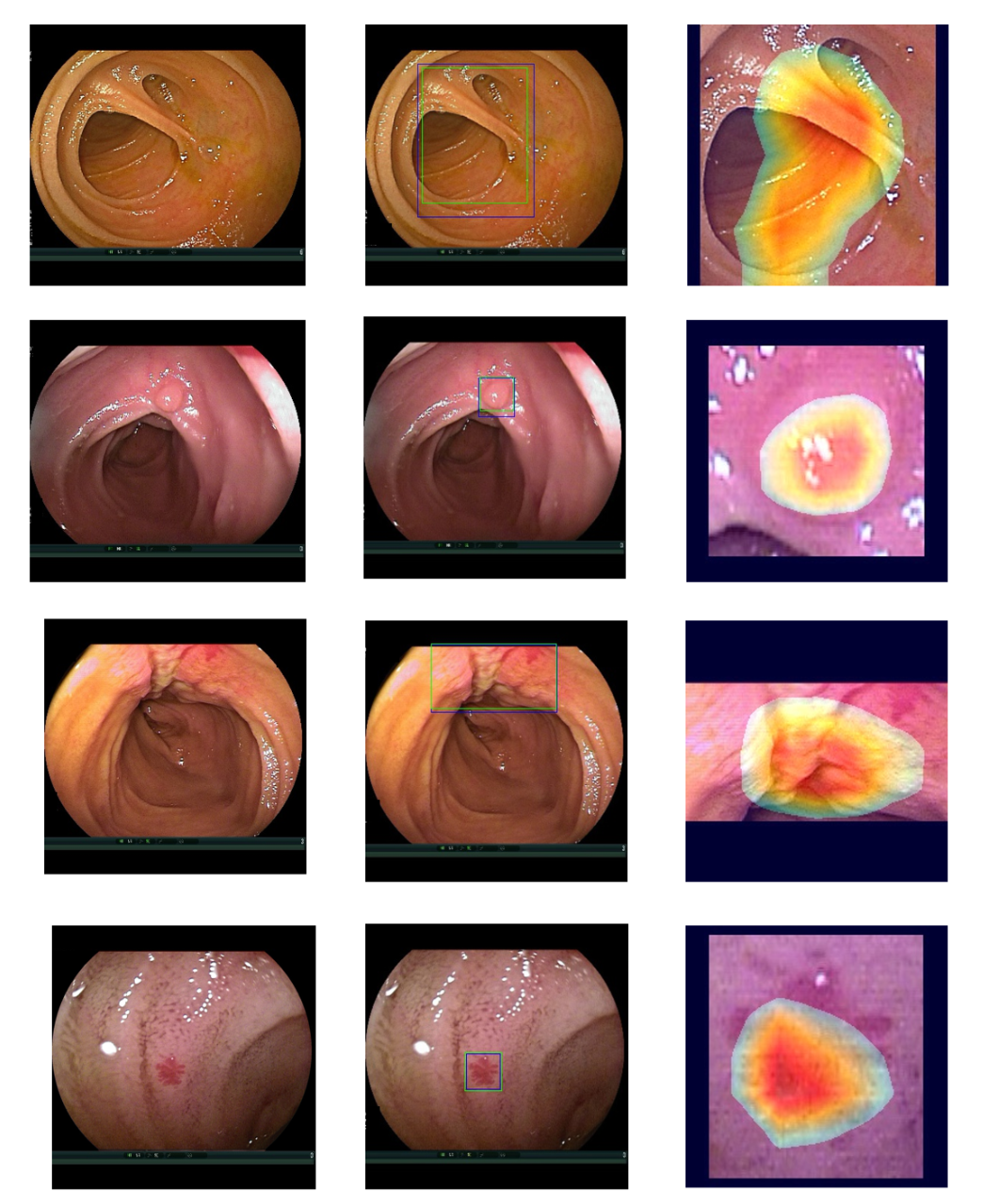


**Supplementary Figure 2. Typical images of four kinds of common diseases.** From the top to bottom is: diverticulum, protruding lesions, erosion & ulcer and angioectasia. The blue box is machine auto-annotation (labeled by the system) and the green box is the standard answer (labeled by experts). The color depth of the heat map is positively correlated with the prediction confidence.


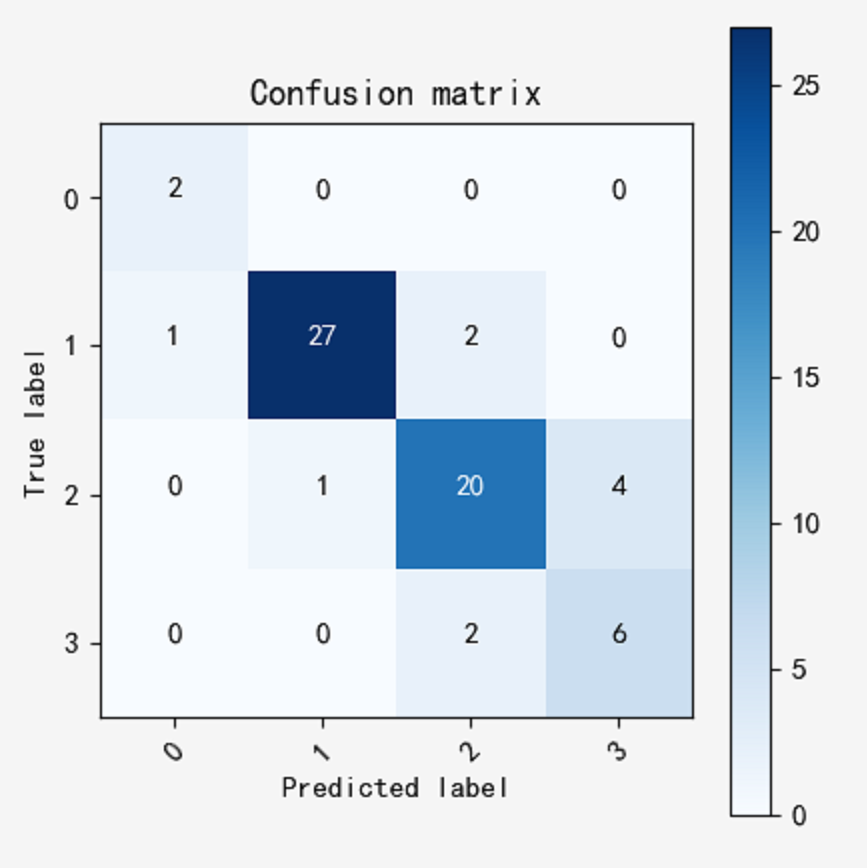


**Supplementary Figure 3. Confusion matrix of ENDOANGEL-DBE in video test set.** “0” represents “diverticulum”, “1” represents “protruding lesions”, “2” represents “erosion& ulcer” and “3” represents “angioectasia”.
